# The Ideal Graduate Study Institution.—What Germany Has Done

**Published:** 1909-07-03

**Authors:** 


### Post-Graduate Medical Section.

### THE IDEAL GRADUATE STUDY INSTITUTION.—WHAT GERMANY HAS DONE.

### 1.—THE CENTRAL COMMITTEE FOR GRADUATE STUDY IN GERMANY.

It is the doctor's task to carry out measures for the preservation of health; the mental and physical labour thus demanded of him is given to serve the well-being of the community.—*Excellenz von Bergmann.*

On May 18, 1900, there was organised in Berlin one of the most important committees for the promotion of post-graduate study in medicine that is to be found anywhere in Europe or America. This committee, which was called into being by a group of enthusiastic members of the medical profession who received the warm and active support of the Prussian Minister for Culture and Education, has since that time done such excellent work and established so excellent a system of graduate study in the whole of the German Empire that it may well serve as a model for other countries. Thus a brief outline of its history and the great progress it has made since its inception must be of interest to everyone who is concerned in the future of graduate study. For the details that follow we are indebted to the courtesy of the Honorary Secretary of the committee, the Director of the Kaiserin Friedrich Haus, Professor Dr. Kutner.

The Central Committee for the Promotion of Graduate Study in Medicine (*Das ZentralKomitée für das ärztliche Fortbildungswesen* *) was formed in order to organise graduate study in Prussia on a sound and businesslike basis, and from its inception two main objects were held in view. These were—

1. To enable practitioners to become acquainted with the advance in medicine and its special branches, and to keep in touch with the practical work which such advance necessitates, by offering them the means and the opportunity for special work and progressive development; and

2. To enable practitioners to increase their knowledge, and thereby to augment their usefulness, without the sacrifice of too much time or money.

In order to achieve these desirable objects in a manner most beneficent to general practitioners the committee recognised from the very first that whatever courses or opportunities were to be offered to practitioners these should be available free of charge, so that no member should be put to any expense, even a nominal one, in attending them; that the courses should be held in the immediate neighbourhood of the practitioner's home, and finally that they should be held at times when it was most convenient for the majority of practitioners to attend them. Here at the very start the committee formulated an ideal to which, it is satisfactory to add, it has steadily adhered. This ideal is that the graduate study of the practitioner is not a matter of weeks or months, but a life-long process. The medical man must remain a student if he wishes to keep abreast of modern advance and modern thought, but it is too much, and in fact entirely unnecessary, to expect him to devote a certain specified time daily or yearly to the scientific study of new developments. The ordinary practitioner is much too busy a man to wade through special monographs and transactions, and specialism has narrowed so greatly of late years, and within its limits each speciality has built up so many vast structures, that it is well nigh impossible for him to grasp the essentials of every new step in medicine. Yet, as Professor von Bergmann so aptly expressed it in the sentence that serves as text to this article, the welfare of the community, no less than the interest of the profession, demands that the practitioner should be acquainted with the recent advances in science and that he should follow the development of his art and science. It was necessary, therefore, to give the practitioner a helping hand, and the State, recognising the importance of the matter, stepped in and lent the movement its cordial support. The ideal, accepted by the State as well as by the profession in Germany, has never been lost sight of, and the result is that graduate study in Germany, so far as German practitioners are concerned, is organised on the soundest basis and on lines which leave little, if anything, to be desired.

It is interesting to notice how the committee achieved this gratifying result. It admitted, from the commencement, that it is wrong and unjust to expect qualified men to resume their places among the unqualified students in order to acquire further knowledge. Many a practitioner who would willingly join a graduate class shrinks, from motives which will be appreciated by every graduate, from joining a "mixed class" of the type which obtains in England. Equally was it evident that teachers who possess a reputation as student teachers will not necessarily do for qualified men; the choice of instructors was, therefore, one of the points which the committee had to consider. Finally, there was the question of time and place. In the large University towns, where clinics and laboratories existed, special arrangements had to be made in order to keep to the ideal of pure graduate classes. All this demanded much careful thought and consideration. The magnificent result of which the committee can be proud to-day was not obtained in a day, but it was obtained and it exists as an example of what may be done elsewhere by organisation and systematisation.

The first duty of the committee was to organise the teaching staff and the clinical material, and in this it had the earnest help of the professors and directors of the large clinics and hospitals. Nor was the movement confined to University towns. The ideal was to establish branches in every city that possessed the clinical material, to found centres for instruction at least in each district. At present there are such branches, at which regular graduate courses are given under the auspices of the Central Committee in no less than 27 Prussian and 28 other German towns. Every year the committee publishes a report which shows a gradual but regular increase in the number of branch centres and in the number of graduate students attending these courses. Nor is this all. These reports also furnish German practitioners with valuable information concerning graduate courses in towns outside the German Empire, and the committee serves as an information bureau as well. Yet another and more important result which has been achieved is the establishment of so-called

#### Medical Academies.

These have been established in non-University towns which possess a large amount of available clinical material, and are therefore specially suited for graduate study. The

* The English equivalent does not possess the happy terseness of the German "Fortbildungswesen," but adequately describes the object of the committee. The real founder of the committee was the late Professor von Bergmann, who, together with several Berlin medical men, organised in 1900 a Society for the Holding of Courses for Practising Physicians, which subsequently adopted its present title on the recommendation of Ministerial Director, Dr. Althoff.

academies at Cologne, Düsseldorf, and the excellent branch at Hamburg, at which centres it is a well-known fact that the best post-graduate courses are given, have been established mainly through the activity of the Central Committee. These academies are in direct connection with the Central Committee. Their medical staffs are connected with the local branch for post-graduate study; their directors are *ipso facto* members of the Central Committee, which has the right to nominate a representative on the board of management of each institution. The result of this triple connection has been a most harmonious co-operation between these institutions and the Central Committee.

The Central Committee, as at present constituted, consists of the Honorary President, Prince von Bülow; three honorary members; of an executive; of extraordinary members representing the Governments of Baden, Bavaria, Saxony, and Wurtemberg; and of ordinary members representing the city of Berlin, the medical societies of Berlin, the committee for the promotion of graduate study in dentistry, the Kultus ministerium, the medical faculty of the University, the committee for medical "Studienreisen," a director of a municipal hospital in Berlin, a director of a non-municipal city hospital, the medical directorate of the Charité, and a representative of the medical profession in the province of Brandenburg; and in addition the presidents of the several local branches. The office of the committee is Luisenplatz 2-4, Berlin, N.W.

When the committee was formed post-graduate courses were only given at irregular intervals at nine centres. To-day regular courses are given at no less than 46 centres in every possible subject, directly or indirectly under the auspices of the committee, and for the most part free. These courses are open to every German practitioner, and are practical and theoretical. Having, through the courtesy of the demonstrators, had an opportunity of attending several of them we can speak from experience of their usefulness. They are in every case arranged to suit the practising physician, to supply his wants and meet his wishes, and, beyond paying two marks as a membership fee, the member who attends such courses has absolutely no expenses, even where the course demands a considerable outlay in instruments and material. The immense educative value of these courses can hardly be over-estimated, and the fact that membership is in every case full shortly after the announcement of each course shows very well how popular they are. From time to time lists of such courses are published. The committee has its own organ—with which we hope to deal later on—and has worked hard and successfully to bring all these opportunities to the notice of practitioners in Germany.

How is it all done? The question of ways and means is always an interesting one, and in this connection it is doubly so. The work has demanded sacrifices and it has cost money. For the first the committee and its supporters have done their share. The professors and teachers have thrown themselves heart and soul into the work, and have had their reward in the success that has attended their efforts. The State and private munificence have seen to the expense, and in many cases the practitioners who have established a local centre have borne their share, though not as members of a course but as private individuals. One of the most remarkable results of the pioneer work of the committee, indeed, has been the enthusiasm and the spirit of emulation it has aroused in other parts of Germany. Practitioners have attended courses away from their sphere of activity, and liked these so much that they have clamoured for and obtained the establishment of local branches. These branches have been encouraged by the Central Committee, which, helped by a donation from the Ministry for Education, has formed the magnificent

State Collection of Demonstration Specimens.

Such is the official title of this interesting department, which was formed with the object of providing the various local centres (four only of which at present possess adequate collections of pathological interest) with material for demonstrations. The primary cost of the arrangement was borne by the Kultus ministerium, but the collection has been greatly increased and its usefulness added to by the gifts of private institutions and the donations of individual benefactors. Thus the late Professor Lassar bequeathed the unrivalled private collection of wax models illustrating diseases of the skin, Dr. Siemerling, of Kiel, presented a representative collection of nerve specimens, while from various other sources came valuable additions. A fair number of necessary models and specimens had to be purchased, and as the collection stands at present it may be looked upon as one of the finest and most comprehensive of its kind. It comprises anatomical preparations, general and special; pathological-anatomical preparations, general and special; microscopical preparations, divided into (*a*) general, (*b*) special, (*c*) pathological, (*d*) clinical, and (*e*) bacteriological; plastic representations or wax models representing (*a*) acute infections, (*b*) chronic exanthems and skin diseases, (*c*) parasitic and non-parasitic diseases, (*d*) eye diseases, (*e*) intestinal diseases; plaster models divided into (*a*) anatomical, (*b*) genito-urinary, (*c*) skin, and (*d*) throat diseases (among these may be seen the model of the laryngeal cancer of the Emperor Frederick); and papier maché preparations divided into (*a*) physiological and (*b*) surgical; books and engravings, photographs, charts and drawings, divided into 27 groups and comprising every possible subject of interest to the physician—a most valuable, and, in some respects, a unique collection, containing as it does the valuable and interesting private collection of medical miscellanea of Professor Hollander; lantern slides—a special department with a very complete collection—and other transparencies; stereoscopic preparations and apparatus; microscopic apparatus; phantoms for demonstration purposes; a very rich collection of kinematographic films, anatomical, physiological, pathological, and clinical, with spare apparatus; and finally, an equally fine collection of bacteriological cultures, permanencies, slides, and projection apparatus.

The collection is in daily use, not only in Berlin but throughout Germany, and if any proof of its usefulness is wanted the Director's day-book, with its large number of entries, is sufficient to show that it is rapidly becoming very popular. Every teacher, every professor or privat-docent who gives a course, whether to graduates or to students, can avail himself of this collection. Let us imagine, for example, that the doctors of a little South German town wish for a course in diseases of the eye. There is, perhaps, one of them who knows his eyework well and who is able to give this course, but under usual circumstances the clinical material available is far too small to permit of regular study. So there comes an application to the Director, "Please send, by return post, models and specimens to illustrate course of ophthalmology." These are sent at once, packed, registered, and catalogued, with histories and full particulars. The applicant pays cost of carriage and insurance and undertakes to return the specimens as soon as he has finished the course. So, too, in other departments. A local doctor is asked to give a course on first aid to the senior pupils of the gymnasium, to deliver a lecture on tuberculosis, or to demonstrate the advisability of vaccination. He, too, sends to the Director and avails himself of the magnificent collection, and is thereby enabled to illustrate his lecture or course in a very efficient and comparatively inexpensive manner. There are models here that have travelled all over Germany, doing useful work wherever they went, and the practical advantages of such a collection on such lines are incalculable. Its educative value is enormous, not merely from the point of view of graduate study, but equally so from that of the popular lecturer. The doctor can do much to influence local thought and opinion in matters of hygiene and public health by means of occasional public and popular lectures. Such occasional excursions will be of value to him as well as to his hearers, and many practitioners will take a larger interest in what is as much their duty as the writing of prescriptions if they were certain of such help as this collection assures the German practitioner. In conclusion, it is merely necessary to add that a full and detailed catalogue of the collection has been printed, which is forwarded on application to every practitioner in Germany. In special cases, the Director assures us, arrangements can be made to enable lecturers outside Germany to avail themselves of the collection as well.

Another feature of the work of the committee has been the development of post-graduate courses in dentistry. These courses are exceedingly popular and their educative value is daily becoming more apparent. Not the least remarkable of the results which have been obtained by this effective organisation is the almost universal recognition in the German Empire of the necessity for such graduate courses, and the interest which has been awakened by the labours of the committee in the profession generally and in the several States. The latter have supported the movement in a most exemplary and praiseworthy manner, and it is not too much to declare that the Central Committee at present is a national asset of the German Empire which does as good work, and is claiming as effective support and obtaining it, as the various Universities that cater for the student and for the medical practitioner up to the time that he is launched into practice.

